# Fellow-Workers and the Roll of Honour

**Published:** 1915-09-18

**Authors:** 


### ROUND THE WORLD.

[The Editor invites Early Information and Contributions Marked "Fellow-Workers."]

### Fellow-Workers and the Roll of Honour.

THE increasing size of our armies abroad necessarily calls for more assistance in all ranks of the medical department, and a steadily lengthening list of consultant appointments indicates the demands made upon the numerous eminent physicians and surgeons who have accompanied our troops to the Front. Amongst recent additions to the roll of specialists acting chiefly in an advisory capacity may be mentioned Mr. Andrew Fullerton and Mr. Alexis Thomson, distinguished representatives of the Irish and Scottish Schools of Surgery respectively, both of whom have been appointed consulting surgeons to the Expeditionary Force in France, with the rank of colonel.

A WELL-KNOWN Belfast surgeon, Colonel Fullerton, as he now is, has for some years held various public appointments of importance in that city, including those of outpatient surgeon to the Royal Victoria Hospital, outpatient surgeon to the Hospital for Sick Children, and consulting surgeon to the Cripples' Home. A scholar of Queen's College, Belfast, he qualified in 1890, and subsequently became an M.D. and F.R.C.S.I. In early years Colonel Fullerton held resident posts at the Miller Hospital, Greenwich, and at the West Kent Hospital, Maidstone.

COLONEL HENRY ALEXIS THOMSON went abroad some little time ago to one of the base hospitals, where, with the rank of major, he has had charge of much responsible surgical work. A brilliant student of both Edinburgh University and the London Hospital, he graduated with honours in 1885, and a few years later took the gold medal at the M.D. Edin. and became an F.R.C.S. Edin., adding to these academic distinctions the M.R.C.S. Eng. and B.Sc. Edin. Colonel Thomson, who has been a prolific writer on various important points in practical surgery, occupies a leading place amongst Scottish practitioners, being surgeon to the Royal Infirmary, Edinburgh, and professor of surgery in the University.

WORK at the five military hospitals in Exeter is occupying a number of medical men, including several distinguished practitioners whose reputation extends far beyond the West Country. The *British Medical Journal* recently published (August 28, 1915) a special account of the war hospitals in Devon, and according to this the medical staffs of the Exeter institutions are constituted as follows: Mr. A. L. Candler, F.R.C.S., is in charge of No. 1, to which Dr. William Gordon, M.D., F.R.C.P., is physician, Dr. G. T. Clapp and Dr. Mabel Gates being also attached.

AT Exeter No. 2 Hospital Dr. R. A. Worthington is M.O. in charge; Dr. Henry Andrews, Dr. Solly, and Dr. Lovely (the last-named as resident) being also on the staff. No. 3 has Dr. S. E. Atkins in charge, with Dr. Henry Davis and Dr. Bingley Pullen visiting. Then No. 4 having been organised for special work under R.A.M.C. officers, it is understood, No. 5 is in charge of Dr. Brennan Dyball, with Dr. Robin in residence, the visiting medical officers being Mr. Marmaduke Shield, Dr. Samways, Dr. Wilton, and Colonel J. Raglan Thomas, M.D.

SPECIALIST work at these hospitals is carried out by expert medical officers, who visit each of them as required. Mr. A. C. Roper is ophthalmologist, Mr. J. Ackland looks after dental cases, Dr. J. D. Harris superintends the radiography department, and Dr. R. V. Solly carries out pathological and bacteriological work, using the special department of the Devon and Exeter Hospital as a central laboratory.

IN the department of pathology at Edinburgh University a band of investigators has been inquiring into the utility of hypochlorous acid regarded as an antiseptic application for wounds, at the request of the Medical Research Committee. As a result of a large number of experiments the observers concerned—Dr. J. Lorrain Smith, Dr. A. Murray Drennan, Dr. Theodore Rettie, and Dr. William Campbell—have come to the conclusion that hypochlorous acid is one of the most powerful antiseptics known, and, according to their published report (*British Medical Journal*, July 24, 1915), has many advantages as a field dressing, or in the local treatment of wounds at any time.

DR. JAMES LORRAIN SMITH, M.D. Edin., F.R.S., is the professor of pathology at Edinburgh, and formerly held a similar post at Queen's College, Belfast, and at the Victoria University, Manchester. His researches have added considerably to our understanding of various physiological processes, amongst which may be specially mentioned the relation between respiration and purification of the blood.

DR. ALEXANDER MURRAY DRENNAN, M.D., F.R.C.P. Edin., who is now professor of clinical pathology in the University of Otago, New Zealand, has done much useful work in the pathological department at Edinburgh, where he has held the posts of assistant pathologist and lecturer on practical pathology. Dr. Drennan, who graduated with first-class honours in 1906, had a brilliant academic career as a student, winning several important scholarships.

DR. WILLIAM CAMPBELL, who now holds a commission as lieutenant in the R.A.M.C., graduated in medicine and surgery with first-class honours at Edinburgh in 1911, and subsequently became appointed demonstrator of pathology there. He has held resident appointments at the Bradford City Hospital, the French Hospital, London, and at the London School of Tropical Medicine.

LIEUTENANT-COLONEL PETER MURRAY KERR, who has lately been invalided home, although commanding a combatant force—the 1/5th Battalion King's Own Scottish Borderers—is in private life a well-known practitioner in Dumfries. Qualifying at Edinburgh University in 1887, Lieutenant-Colonel Kerr, on settling in practice, became eventually senior surgeon to the Dumfries and Galloway Royal Infirmary, and a J.P. for the district.

CAPTAIN JAMES JOHNSTONE DYKES, lately killed in the Dardanelles, was another Dumfries professional man, and attached to Lieutenant-Colonel Murray's battalion of the King's Own Scottish Borderers; he was himself likewise possessed of medical and surgical qualifications. In addition, he held the dental diploma of Edinburgh University, and was honorary dental surgeon to the Dumfries and Galloway Royal Infirmary, as well as to a large local industrial school.

